# Perthes Syndrome associated with intramedullary spinal cord hemorrhage in a 4-year-old child: a case report

**DOI:** 10.1186/1757-1626-1-17

**Published:** 2008-06-13

**Authors:** Mehmet Senoglu, Nimet Senoglu, Hafize Oksuz, Gokhan Ispir

**Affiliations:** 1 Department of Neurosurgery, Kahramanmaras Sutcu Imam University, Kahramanmaras, Turkey; 2 Department of Anesthesiology and Reanimation, Kahramanmaras Sutcu Imam University, Kahramanmaras, Turkey

## Abstract

**Background:**

Perthes Syndrome (Traumatic asphyxia) is rare, which is caused by sudden compressive chest trauma and characterized by subconjunctival hemorrhage, facial edema, craniocervical cyanosis, and petechiae on the upper chest and face.

**Case presentation:**

We present the case of a 4-year-old Caucasian girl who developed traumatic asphyxia associated with intramedullary spinal cord hemorrhage following thoracic compression.

**Conclusion:**

We have not found the association of Perthes syndrome with intramedullary spinal cord hemorrhage described in the medical literature. To our knowledge, the current case is the first report of Perthes Syndrome associated with intramedullary spinal cord hemorrhage.

## Background

The acute thoracic compression syndrome has been variously termed; ecchymotic mask, traumatic asphyxia and Olivier's or Perthes' syndrome, among others [1].

Traumatic asphyxia is a rare syndrome, first described over 150 years ago by Olivier [2]. It is caused by sudden compressive chest trauma and characterized by subconjunctival hemorrhage, facial edema, craniocervical cyanosis, and petechiae on the upper chest and face. Traumatic asphyxia is usually of little prognostic significance, although associated injuries may be life-threatening [3-8].

Pathophysiological aspects of traumatic asphyxia in children differ from that seen in adults because of the greater elasticity of the thorax in children [9]. According to our knowledge, Perthes Syndrome associated with intramedullary spinal cord hemorage has not been previously reported in the medical literature. We present a 4-year-old child developing traumatic asphyxia associated with intramedullary spinal cord hemorrhage fallowing by thoracal compression.

## Case presentation

**Figure 1:**
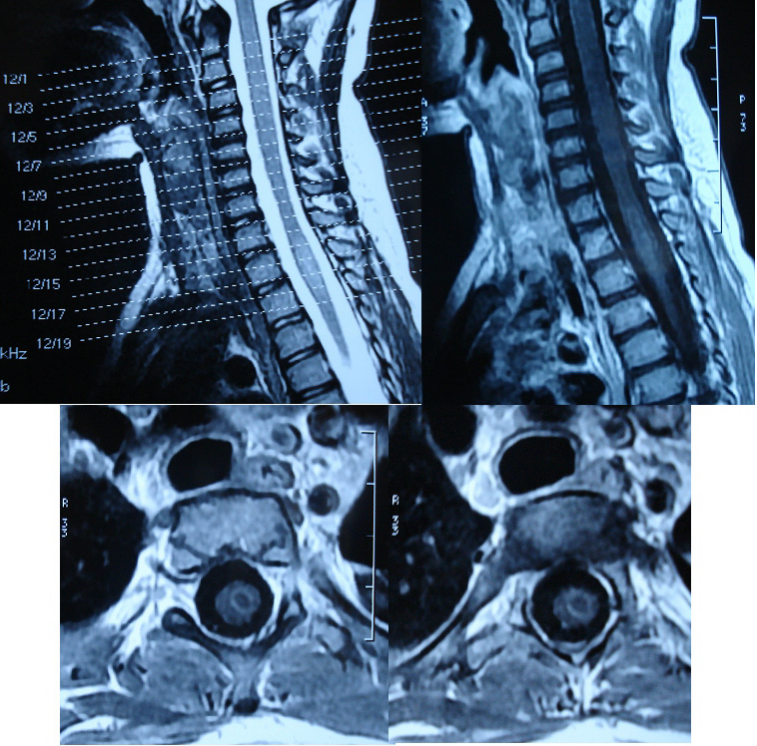
**Cervical MRI: An intramedullary hemorrhage lesion is seen at the the Th 2–3 intervertebral disc level situated on the Cervical MRI**. The lesions were heterogeneous on Th1 and Th2 weighted images.

A 4-year-old, Caucasion, girl was admitted to the emergency service in an unconscious state after a cardiopulmonary resuscitation in another hospital. She had compression of the whole of the left arm, the shoulder and the left side of the thorax, caused by a lift. Upon arrival in the other hospital, about 15 min after the accident, she had cardiopulmonary arrest, intubated orotracheally, cardiopulmonary resuscitation was performed for 15th minutes. Her head, neck, and upper chest were strikingly cyanotic and edematous, with multiple petechiae; she also had bilateral subconjunctival hemorrhages. Her neck was stabilized with a hard collar. In neurological examination; the patient was Glasgow Coma Score of E1M2Ve [coma score of 3], papillary was isochoric and light reflex were bilaterally reactive. She was paraplegic. Past medical history was noncontributory. Her heart rate was 130 bpm, arterial blood pressure was 100/65 mm Hg, and peripheral pulses were weakly palpable in both upper extremities. Breath sounds were not obtained in the left hemi thorax. The abdomen was soft. Blood gas analysis just after she was admitted to the intensive care unit was: pH 7.30, PaCO2 45 mmHg, PaO2 60 mmHg, base excess -2.8 mMol·L-1, breathing 5 L·min-1 100% oxygen by self-inflating bag via endotracheal tube. On the pulmonary x-ray, pneumothorax was determined and tube thoracostomy was performed. Spinal and abdominal X-ray and echography showed no injuries. Hemorrhage and edema were not observed in the brain and spinal by computed tomography. Ophthalmological examination, including fundoscopy, revealed no abnormalities. Respiratory failure developed and the patient required mechanical ventilation. She was observed in the intensive care unit and treated with methylprednisolone sodium succinate within 24 hours (intravenous bolus of methylprednisolone [30 mg/kg], methylprednisolone infusion of 5.4 mg/kg per hour for 24 hours). After the respiratory failure was recovered, on the 20th day, cervical Magnetic Resonance Imaging revealed an intramedullary hemorrhage lesion to Thoracal [Th ] 2–3 intervertebral disc level situated. The lesions were seen heterogeneous on Th-1 and Th-2 weighted images, which is started to resorption [figure 1]. The patient transferred to the rehabilitation unit in state of Glasgow Coma Score of E3M5V2 [coma score of 10].

## Discussion

Traumatic asphyxia is usually caused by severe thoracic compression but has also been associated with asthma, paroxysmal coughing, protracted vomiting, and jugular venous occlusion [10].

Cases of traumatic asphyxia are mainly a consequence of motor vehicle crashes. Other causes include heavy machines, furniture and, rarely, deep-sea diving, epileptic seizures, difficult delivery and asthmatic attack [2]. In literature, the typical range of the duration of compression is between two and five minutes [11]. The duration and the weight of compression affect the outcome following traumatic asphyxia. Considerable weight can be tolerated for a short period, whereas a comparatively modest weight applied for a longer period may result in death [1,2,11,12]. In our case, the duration of compression was about five minutes.

After traumatic asphyxia, patients often demonstrate striking physical findings, including blanching erythema and petechiae of the head and neck, as well as cervicofacial edema. Often there are transient neurological symptoms, such as blurred vision and hearing loss [13].

In the present case, there was paraplegia. Perthes Syndrome associated with paraplegia has not been previously reported. Williams et al. [13] studied the effects of chest compression on intrathoracic venous pressure in an animal model. When the glottis was open, only a small rise in jugular venous and superior vena cava pressures was observed. A significant increase of venous pressure occurred only when the glottis was closed during chest compression. The cutaneous findings of traumatic asphyxia are believed to result from an increase in capillary pressure, which causes rupture of small vessels, petechial hemorrhages, and hydrostatic edema [14]. The vision can be affected by the same mechanism; for example, retinal hemorrhage has been associated with severe chest compression (Purtscher's retinopathy). In addition, a hearing deficit can be caused by edema of the eustachian tubes [3,10,13-15]. Severe abdominothoracic compression – or purely abdominal compression in some cases – can cause acute, high-pressure venous stasis. Stasis affects the avalvular network of both venae cavae, particularly the superior. It results in capillary dilatation and, after several minutes, haemorrhagic suffisions under the skin [1].

Cervical venous drainage is provided by the internal and external jugular veins. The tissues and structures beneath the deep cervical fascia (i.e., larynx, trachea, oropharynx) drain into the internal jugular vein while those above it [subcutaneous tissue of the scalp and neck) drain into the external jugular vein. Although the external jugular vein has two pairs of valves, they are not competent to prevent flow reversal [16]. In contrast, the brain, the deep soft tissues of the neck, and the airway are protected against reversal of venous blood flow by valves in the internal jugular vein; however, these valves may also become incompetent at pressures exceeding 45 mm Hg [17]. This may explain why the scalp and superficial cervical tissues are more likely to be affected than the brain and airway in traumatic asphyxia [3,16-18].

The infrequency of cerebral hemorrhage is explained by the support given to the blood vesels of the brain and meninges by the intracranial pressure, analogous to the support of the retinal vessels by the intra-ocular pressure. The severity of edema in this syndrome varies from involvement of the subcutaneous tissue to swelling of the deep tissues. Mild to moderate cervical prevertebral soft tissue swelling with dysphagia, hoarseness, swollen tongue, and cervical hematoma has also been observed [18]. Although cord injury has not been described with venous hypertension from crush injury it, there have been descriptions of cord dysfunction with other causes of increased venous pressure. In the present case, our opinion that spinal cord can be affected by the increased venous pressure and intrameduller spinal hemorrhage developed at the Th 2–3 level. It is important to know that in unconscious cases, who had traumatic asphyxia, venous and capillary pressure increases; this pathopyhsiological process may result in spinal hemorrhage [3,16-18].

Traumatic asphyxia may also be complicated by serious thoracic injuries including pneumothorax, hemothorax, rib fractures or flail chest, and mediastinal injuries, and head injuries. It is reported that mortality rate is usually low in children because they have elastic chest cage [19]. In the presented case pneumothorax was determined and tube thoracostomy was performed.

## Conclusion

The case presented here is the first described in the medical literature which result from Perthes Syndrome. It is important to the clinicians to be aware of the spinal cord hemorrhage can be accompanied to the traumatic asphyxia and treating with steroid immediately after the trauma without radiological evidence.

## Abbreviations

MRI: Magnetic Resonans Imaging; Th: Thoracal

## Authors' contributions

MS carried out the patient's diagnosis, drafted the manuscript, NS performed the case management, drafted the manuscript, HO participated in the patient's management, GI performed the clinical work. All authors read and approved the final manuscript.

## Consent

Written informed consent was obtained from the patient's father for publication of this case report and accompanying images. A copy of the written consent is available for review by the Editor-in-Chief of this journal".
